# Beyond the Brain: The Physical Health and Whole-Body Impact of Fetal Alcohol Spectrum Disorders

**DOI:** 10.35946/arcr.v45.1.05

**Published:** 2025-06-12

**Authors:** Chelsea Vanderpeet, Lisa Akison, Karen Moritz, Nicole Hayes, Natasha Reid

**Affiliations:** 1 Child Health Research Centre, The University of Queensland, Brisbane, Australia; 2 Department, School of Biomedical Sciences, Faculty of Medicine, The University of Queensland, Brisbane, Australia; 3 School of Early Childhood and Inclusive Education, Queensland University of Technology, Brisbane, Australia

**Keywords:** alcohol, prenatal alcohol, fetal alcohol spectrum disorders, prenatal exposure delayed effects, sleep, metabolism, cardio-renal, vision

## Abstract

**PURPOSE:**

Individuals with fetal alcohol spectrum disorders (FASD) or neurodevelopmental disorder associated with prenatal alcohol exposure (PAE) can experience a wide range of whole-body health conditions. A survey by the International Adult Leadership Collaboration (ALC) FASD Changemakers found that many adults with FASD have comorbidities relating to metabolic disorders; body composition; cardio-renal, reproductive, and/or immune health; as well as difficulties with hearing/vision and sleep. This review summarizes current knowledge of these health domains and provides an overview of the latest literature on the whole-body effects of PAE/FASD across the life span.

**SEARCH METHODS:**

The literature search was conducted on July 8, 2024, using CINAHL, PubMed, and Web of Science databases. To investigate the whole-body health of individuals with PAE, search terms were based on the findings of the ALC FASD Changemakers Health Survey and covered areas relating to sleep; hearing/vision; body composition; and metabolic, cardiovascular, renal, immune, and reproductive health. The search was conducted in two phases. To summarize current knowledge on these topics, the latest systematic reviews and other reviews were identified for each health domain (phase one). In addition, recent primary research articles published since these review searches were completed were identified for each domain (phase two). Inclusion/exclusion was based on article relevance to the physical health challenges reported in the ALC FASD Changemakers Health Survey.

**SEARCH RESULTS:**

In phase one, 744 reviews were identified in the initial search, of which 722 articles were excluded and 22 recent and relevant reviews were included. In phase two, 1,102 articles were identified, with 665 screened at the title/abstract level and 169 articles undergoing full-text review. A total of 1,066 articles were excluded. Following the addition of five articles from other sources, 41 recently published primary articles were included in the current review.

**DISCUSSION AND CONCLUSIONS:**

A growing body of evidence suggests that individuals with PAE/FASD may experience comorbidities relating to metabolism; body composition; cardio-renal, immune, and/or reproductive health; as well as hearing, vision, and sleep difficulties. These findings support the concept of FASD as a whole-body diagnosis, emphasizing the importance of a holistic approach that supports the overall health and well-being of those with PAE. There are opportunities for future clinical research to focus on further understanding these physical health challenges, how they evolve, and how effective intervention approaches could improve outcomes for individuals with PAE/FASD.

Prenatal alcohol exposure (PAE) can be associated with an array of physical, developmental, and behavioral effects.^1^ Internationally, there is no agreed-upon set of diagnostic criteria for individuals who have PAE, and a range of diagnostic terminologies are currently in use. Broad terminologies that include the range of physical and neurodevelopmental effects include fetal alcohol spectrum disorders (FASD) and neurodevelopmental disorder associated with PAE.^2^–^5^ Additionally, some criteria further differentiate diagnostic outcomes based on the presence or absence of physical features (e.g., fetal alcohol syndrome, partial fetal alcohol syndrome, static encephalopathy, neurobehavioral disorder, or alcohol-related neurodevelopmental disorder).^6^–^8^ The global pooled prevalence of FASD is estimated to be 7.7 per 1,000, with rates in some at-risk subpopulations (e.g., children in out-of-home care and correctional settings) estimated to be 10 to 40 times higher.^9^,^10^ The degree of fetal alcohol effects varies widely among individuals and is influenced by the dose and timing of PAE as well as other environmental and genetic factors.^11^ This variability is reflected in the heterogeneity of outcomes and FASD diagnostic criteria and categories.^12^

Popova et al.^13^ have highlighted the high prevalence and wide variety of comorbid conditions in individuals with FASD/PAE. A large body of evidence has examined the adverse neurodevelopmental and mental health outcomes that individuals with PAE can experience.^14^,^15^ The profound social and economic effects associated with FASD are also relatively well documented.^15^,^16^ In comparison to these areas, research on the relationship between PAE and physical health outcomes is more limited.

Whereas environmental factors are thought to play a significant role in the health of individuals with FASD, there is a modest amount of preclinical studies indicating that alcohol exposure in utero can contribute to physical health difficulties later in life. A growing body of clinical evidence is linking PAE to a wide variety of physical health challenges.^17^–^24^ These findings align with the Developmental Origins of Health and Disease hypothesis proposed by Barker et al.,^25^ which suggests that in utero stressors may have a lasting impact on long-term health. This hypothesis is often viewed in the context of a “dual strike,” where the initial PAE makes the individual vulnerable to other environmental stressors later in life. Therefore, it is reasonable to propose that the teratogenic effects of PAE could also contribute to physical health and the development of chronic disease, which may further be compounded by a range of adverse postnatal experiences. This has significant implications for the overall health and well-being of individuals with PAE/FASD.

In 2020, a survey titled “The lay of the land: FASD as a whole-body diagnosis” by the International Adult Leadership Collaboration (ALC) FASD Changemakers, hereafter referred to as the FASD Changemakers Health Survey, further emphasized that the impact of PAE extends far beyond childhood and the standard physical features (i.e., facial dysmorphology and physical size restriction) and neurobehavioral effects commonly focused on in research and clinical practice.^26^ This survey included more than 500 adolescents and adults with FASD, ranging in age from 16 and younger to 60 and older. It captured self-reported health data from across a variety of domains, meaning that it only reflects individuals’ awareness of their own health and may not show all possible conditions. Additionally, poor access to health care and asymptomatic conditions may contribute to underreporting of health problems. Despite these limitations, the FASD Changemakers Health Survey has been a seminal publication, providing insight into the personal physical health experiences of individuals with FASD. The survey highlighted a broad range of persistent and evolving health conditions, including but not limited to, comorbidities relating to metabolic health; body composition; cardio-renal, reproductive, and immune health; as well as difficulties with hearing/vision and sleep. A number of systematic reviews have examined the existing preclinical and clinical evidence on these topics.^17^–^23^ However, there is an opportunity to integrate these findings in a way that offers a holistic picture of the whole-body effects of PAE across the life span.

The ALC FASD Changemakers continue to strongly advocate that: “Research into health outcomes and issues of individuals with FASD is urgently needed to broaden the conversation and provide a fuller picture of FASD: it is not just a brain, behavior, or mental health issue, but it is a ‘whole-body diagnosis’.”^26^^(p212)^ This statement is a powerful reminder to scientists and clinicians that the perspectives and needs of individuals with FASD should be at the forefront of research and health care. Therefore, this review will examine the available literature on the whole-body effects of PAE across the life span, with a focus on summarizing the latest scientific evidence underpinning reported physical health conditions frequently experienced by individuals with FASD, according to the FASD Changemakers Health Survey. A key objective of this review is to amplify the lived experiences of individuals with FASD and highlight the importance of health care that takes a whole-body health and well-being approach for those exposed to alcohol in utero.

## Search Methods

All literature searches were conducted on July 8, 2024, using the CINAHL, PubMed, and Web of Science databases. The search terms were based on some of the health domains reported in the FASD Changemakers Health Survey^26^ and are provided in Table 1. Outcomes included in the diagnostic criteria for FASD (e.g., physical size and dysmorphology) were not included because they have been recently summarized in a systematic review and meta-analysis by Akison et al.^1^ All searches included terms relating to PAE and FASD. An individual search was conducted for each health domain and included keywords related to sleep, hearing/vision, metabolic disease, body composition, cardio-renal health, immune health, or reproductive health.

The searches were performed in two phases. In the first phase, individual searches were conducted for each health domain to identify systematic reviews and other on-topic reviews. This search was done to aid in summarizing current knowledge on these topics and was limited to reviews published between January 1, 2015, and July 8, 2024. For each health domain, the latest relevant review (preferably systematic) was identified and its latest search date used in phase two (Table 1). In the second phase, individual searches were conducted for each health domain to identify recently published primary articles. This search was done to ascertain the latest scientific evidence on these topics. The search was limited to primary articles published since the latest search date of the previously selected review. This was September 1, 2018, for cardio-renal health; October 1, 2018, for metabolic disorders, body composition, and immune and reproductive health; September 1, 2019, for vision; March 1, 2021, for hearing; and September 27, 2022, for sleep.

All references were imported to EndNote X9.3.3 for review at the title/abstract and full-text level. Articles were included if they examined relevant health challenges (as reported in the FASD Changemakers Health Survey) in individuals with PAE or an FASD diagnosis of any age.^26^ Non-peer-reviewed original research or reviews (e.g., conference abstracts) and non-mammalian animal studies were excluded. Preclinical rodent models were included at the authors’ discretion and were dependent on the availability of clinical evidence.

## Search Results

**Figure 1:**
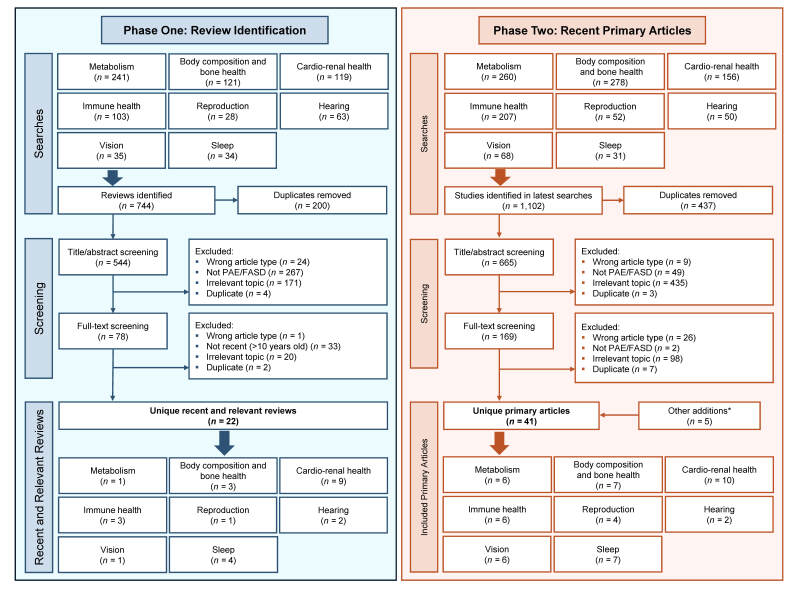
Flow chart of the screening process for this review Individual searches were conducted for each health domain. Phase one was used to identify systematic reviews and other reviews to aid in summarizing current knowledge on these topics. The latest relevant review for each health domain was identified, and their last search date used in the phase-two searches. Phase two was used to identify recently published primary articles to ascertain the latest scientific knowledge on these topics. For both phases, the number of studies in the individual health domains do not add up to the total unique articles included, because some articles reported on more than one health domain. *Two studies were identified from readings and three studies were from other individual health domain searches.

For phase one, the initial searches identified 744 reviews (Figure 1). After removal of duplicates and studies that did not meet inclusion criteria, 22 recent and relevant reviews were identified. For phase two, a total of 1,120 recently published primary articles were identified in the initial searches (Figure 1). After removal of duplicates (*n* = 437), 665 articles were screened at the title/abstract level and 169 articles at full-text level. A total of 629 articles were excluded. Five additional articles were included: two from reference lists and three previously excluded from certain health domain screenings but relevant to other areas explored. A total of 41 recently published primary articles were included. Final numbers of articles included for each health domain were six for metabolic disorders,^27^–^32^ seven for body composition and bone health,^27^,^29^,^33^–^37^ 10 for cardio-renal health,^27^,^35^,^38^–^45^ six for immune health,^21^,^27^,^46^–^49^ four for reproduction,^48^,^50^–^52^ eight for hearing/vision,^27^,^53^–^59^ and eight for sleep.^60^–^66^ The screening process for each health domain is shown in Table 2.

## Results of the Reviewed Studies

### Metabolic Disorders

The FASD Changemakers Health Survey reported high rates of metabolic disorders and associated risk factors in adolescents and adults with FASD.^26^ While the prevalence of type 2 diabetes mellitus (T2DM) was similar between adults with FASD and the general population, impaired glucose metabolism in the form of “hypoglycemia independent of T2DM” was reported by 31% of respondents. Hypothyroidism was reported by 6% of adults with FASD, which is 186 times higher than the general population.^26^ Clinical studies examining the impact of PAE on metabolic disorders related to glucose and lipid metabolism were limited. A systematic review of available literature showed that clinical evidence (*n* = 1 study) suggested some level of impaired glucose metabolism in children with FASD and that preclinical models (*n* = 18 studies) also frequently reported altered metabolism and endocrine profiles.^17^ There is an even greater lack of literature examining PAE and thyroid function.

While there were no recent studies examining clinical markers of metabolic disorders in children and adolescents with PAE, Reid et al.^27^ explored metabolic disorder prevalence through a caregiver questionnaire. They found no difference in rates of diabetes mellitus between the general population and children and adolescents with FASD; however, 5% of caregivers did report clinically diagnosed pre-diabetes. The prevalence of thyroid problems was significantly higher in children and adolescents with FASD (3%) when compared to the general population (less than 1%), indicating that thyroid disorders may be more common in this population from childhood.

Two recent articles examined glucose and lipid metabolism in adults with FASD.^28^,^29^ One study found that there was no difference in fasting blood glucose or hemoglobin A1c in African American adults (approximately age 36) with and without PAE. However, those with PAE and a high body mass index (BMI) had a greater relative risk of diabetic disease when compared to those without PAE at the same BMI.^28^ A larger study by Weeks et al.^29^ found that when adjusted for BMI, adults (ages 18 to 78) with FASD had higher rates of T2DM (12%) relative to the control group (4%). Additionally, 32% to 35% of adults with FASD had abnormal markers of lipid metabolism, twice as many as in the control group.

An additional point of interest is growing evidence of the importance of biological sex in the manifestation of metabolic disorders in this population. While Weeks et al.^29^ reported that adults with FASD were more likely to present with T2DM and markers of abnormal lipid metabolism, they also showed that males with FASD had a higher incidence of these metabolic abnormalities than females, despite having a lower BMI. Additionally, Flannigan et al.^30^ found that while males and females with FASD experienced the same total number of physical health conditions, females had a significantly higher rate of endocrine diseases, including diabetes mellitus and thyroid disorders. These sex-based differential effects of PAE on metabolism and the endocrine system have also been observed across multiple preclinical rodent models.^17^,^31^,^32^ Overall, the literature on metabolic disorders in individuals of any age with PAE is markedly limited. The lack of literature examining thyroid disorders in this population is particularly concerning, because abnormal thyroid activity can have a profound impact on physical and cognitive function.^67^ The existing evidence indicates that individuals with PAE/FASD may have an increased risk of metabolic disorders.

### Body Composition and Bone Health

The FASD Changemakers Health Survey reported a high prevalence of weight-related problems in adolescents and adults with FASD, with over 50% of respondents being either underweight or overweight.^26^ Literature suggests that infants, children, and adolescents with PAE can have low weight and small stature, with the degree of impact likely dependent on the extent of alcohol exposure and FASD presentation.^1^ Physical size parameters have recently been summarized in a systematic review^1^ and thus will not be discussed further in this review. Other systematic reviews showed that this outcome likely is also influenced by age and sex, with females more likely to be classified as overweight after the onset of puberty.^17^,^23^ Clinical studies examining other factors of body composition, such as fat and lean mass, are limited.

Recent clinical articles examining weight and body composition in individuals with PAE have reported variable results.^27^,^29^,^33^–^36^ When examined as a single FASD category without adjustments of confounders (e.g., age and sex), there were no reported differences in rates of parent-reported abnormal BMI between children and adolescents with FASD and the general population.^27^ By contrast, studies that considered age and/or sex tended to observe differences in weight and body composition.^34^–^36^ For example, Hayes et al.^34^ found that adolescent females (ages 12 to 13) with heavy PAE (70 g or more of alcohol per week, at least once per week) had significantly higher BMI z-scores (i.e., differed more from the mean BMI of a reference population) and rates of overweight/obesity relative to typically developing (TD) controls, whereas there was no difference for adolescent males. Smaller studies have explored fat and lean mass in children and young people with PAE, providing some evidence of variable body composition.^35^,^36^ While potentially underpowered, their analyses also indicated that age-dependent and sex-specific effects on body composition may exist in this population.^35^,^36^ The only recent study to examine weight in adults (ages 18 to 70) with FASD found that a significantly greater proportion of females were classified as overweight/obese, while males were significantly more likely to be underweight relative to the comparison group.^29^ This effect was not observed when males and females were combined into a single FASD category. These recent findings highlight the complexity of body composition measures and reinforces the importance of considering sex and age effects in research.

The FASD Changemakers Health Survey reported high rates of bone- and joint-related problems in adolescents and adults with FASD.^26^ Chronic joint problems were twice as prevalent in adolescents and adults with FASD than in the general population, with 22% of individuals with FASD also reporting a mineral density-related diagnoses.^26^ Literature examining these factors is limited in this population, but some clinical and preclinical evidence suggests that PAE may disrupt fetal bone development and contribute to bone- and joint-related health difficulties.^15^

Some recent literature examined bone and joint health in individuals with PAE/FASD. A birth cohort analysis by Parviainen et al.^37^ found that PAE significantly increased the odds of childhood bone fractures independent of potential confounders. Additionally, Young et al.^36^ found that bone mineral density was significantly lower in adolescents (ages 10 to 15), but not children (ages 5 to 9) with FASD when compared to a TD control group. A caregiver report by Reid et al.^27^ was the only recent article to address joint issues, with joint pain reported in 38% of children and adolescents with FASD. Overall, clinical evidence suggests that body composition and potentially bone/joint health is impacted in individuals with PAE/FASD. Clinical research exploring body composition measures beyond BMI is notably limited, which is concerning given the influence of fat and muscle mass on other aspects of physical health, such as metabolism.^68^ Evidence about bone and joint health is also lacking. How these health outcomes may evolve across the life span for individuals with FASD remains unclear, but reports from adolescents and adults with FASD indicate that difficulties documented in children with PAE/FASD may persist into adulthood.^26^

### Cardio-Renal Health

The FASD Changemakers Health Survey reported that adolescents and adults with FASD frequently experienced cardiovascular problems, with rates 1.5 to 20 times higher than in the general population. Heart murmur was the most prevalent cardiac condition reported by adolescents and adults with FASD (21%), and higher incidences of hypertension, coronary heart disease, cardiomyopathy, and heart attack were also documented.^26^ The presence of cardiovascular problems in some individuals with FASD is largely consistent across literature. Relative to other physical health domains, the teratogenic effects of PAE on cardiac development and its potential association with congenital heart defects (CHD) is well explored. There is some variability in the existing evidence, but reviews tend to paint a picture of enduring cardiac dysfunction and vascular abnormalities in some individuals with PAE/FASD.^18^,^24^,^69^–^74^ Studies examining cardiac health beyond structural abnormalities are less common, but some clinical evidence suggests that PAE can impact resting heart rate and cardiac autonomic function.^18^,^45^,^72^,^73^

Four recently published articles assessed rates of CHD amongst infants, children, and adolescents with PAE and FASD.^27^,^38^–^40^ Harvey et al.^39^ examined 5.8 million live newborn singletons and reported that maternal alcohol intake as coded according to the World Health Organization’s International Classification of Diseases was associated with a significantly increased risk of CHD in infants. Similarly, medical records and caregiver reports have indicated that CHD among children and adolescents diagnosed with FASD is high, with rates ranging from 8% to 19%.^27^,^40^ A small study by Beaulieu et al.^35^ was one of the first to examine structural and functional abnormalities through cardiac magnetic resonance imaging. Although the study found no difference between adolescents and young adults (ages 10 to 23) with and without PAE, further exploration in a larger cohort with participants who show facial dysmorphia (i.e., one to three of the sentinel facial features associated with PAE) may help improve understanding of the association between PAE and heart structure and function later in life. Overall, there is some evidence to suggest that individuals with PAE may be at risk of CHD and other cardiac complications. However, literature surrounding cardiac health beyond CHD and the impacts of PAE on the development of cardiovascular disease is limited.

In the FASD Changemakers Health Survey, reports of hypertension were twice as high in adolescents and adults with FASD than in the general population.^26^ Recent literature examining children and adolescents with FASD has also found high rates of hypertension.^27^,^41^ A caregiver report by Reid et al.^27^ found that the incidence of high blood pressure was significantly higher in children and adolescents with FASD (6%) relative to children in the general population (0.2%). Similarly, an exploration of a clinical database by Cook et al.^41^ reported that children and adolescents with fetal alcohol syndrome (FAS)/partial FAS (ages 8 to 17) had significantly greater odds of hypertension compared with data collected from non-FAS/partial FAS individuals in the National Health and Nutrition Examination Survey. There is limited clinical research examining early onset hypertension in individuals with PAE/FASD. This leaves a critical gap in understanding how hypertension may impact broader health outcomes and risk of future cardiovascular/renal complications in this population.

The FASD Changemakers Health Survey reported kidney problems in 10% of adolescents and adults with FASD, five times higher than rates within the general population.^26^ Some preclinical and emerging clinical evidence suggests that PAE may contribute to urinary system defects and altered renal function in this population.^18^,^71^,^75^ Studies examining renal outcomes, especially in adults, remain limited.

Four recently published studies have provided further insight into kidney health and renal function in individuals with FASD.^27^,^42^–^44^ For children and adolescents with FASD, 5% of caregivers reported diagnosed kidney problems (structural or functional), significantly higher than the rate observed in the general population (0.2%).^27^ Dylag et al.^42^ found significantly higher rates of renal and urinary tract abnormalities in children and adolescents (ages 0 to 17) with FASD relative to the TD comparison group. This study also observed significantly smaller kidney length in children and adolescents with FASD, hypothesizing that nephron number may be reduced. When Correia-Costa et al.^43^ examined renal function in children with and without PAE (ages 7 to 8), they found that overweight/obese children with PAE exhibited reductions in estimated glomerular filtration rate that were dependent on the level of PAE. The only recent study to examine renal health in adults with PAE (age 30) found an increased incidence of mild-to-moderate chronic kidney disease relative to the comparison group.^44^ Analyses revealed a dose-dependent effect, with moderate-to-heavy PAE (undefined) associated with the highest odds of chronic kidney disease. Collectively, emerging clinical evidence suggests that renal abnormalities may be more common in individuals with PAE/FASD. Taken together with the reports from the FASD Changemakers Health Survey, these findings suggest that kidney problems may exist across the life span.^26^

Overall, existing evidence suggests that individuals with PAE/FASD may be at risk of cardiovascular and renal problems. The young age when these conditions may manifest is of particular concern, given their potential to impact other aspects of physical health. It is important to note that chronic kidney disease can develop in the presence or absence of congenital defects and may be influenced by additional risk factors such as hypertension.^76^ Distinguishing these two pathways may aid understanding of renal health in this population. Additionally, how these cardio-renal problems may evolve across the life span and influence the development of disease in other systems in individuals with PAE/FASD also remains unclear.

### Immune Health

The FASD Changemakers Health Survey reported high rates of immune dysfunction in adolescents and adults with FASD.^26^ They described higher rates of infection relative to the general population, with 17% to 61% reporting conditions such as chronic/acute sinusitis, ear, kidney, and/or bladder infections. Asthma (36%), eczema (28%), and autoimmune diseases (35%) were also frequently reported.^26^ In the literature, there is limited and variable clinical evidence of the effects of PAE on immune function.^19^,^77^,^78^ A systematic review (*N* = 12 studies) of existing clinical research suggested that PAE may increase the risk of infection and atopy in newborns and children.^19^ Preclinical studies have found some evidence of autoimmune disease and abnormal immune markers (e.g., immune cell parameters and inflammatory mediators), but exploration of these factors in clinical studies is less common.^19^,^49^

Recent literature is similarly variable regarding immune function in children and adolescents with PAE/FASD.^21^,^27^,^46^ Examination of medical records in one study found no difference in the rates of hospital presentation for respiratory infection in the first year of life between children with and without low-level PAE (defined as an average of 6.5 standard drinks over the entire pregnancy).^46^ A caregiver report examining a broader range of immune outcomes found that 27% to 35% of children and adolescents with FASD experienced asthma or skin conditions. Additionally, chronic infections and autoimmune disease were reported by 21% and 2% of caregivers, respectively.^27^ Recent literature also suggests that persistent ear infections are frequently experienced by children and adolescents with PAE.^21^,^27^ These variable findings are not unexpected given the diverse range of conditions included in this domain. Diversity in the PAE amount/timing and the extent of FASD presentation also likely contribute to the variability.

A recently published clinical longitudinal study by Bodnar et al.^47^ is one of the first to examine cytokine profiles in children with and without PAE (ages 2 to 4). They found that children with PAE had a pattern of inflammatory marker activation distinct from children without PAE. Additionally, within the PAE group, cytokine activity differed based on the presence or absence of a neurodevelopmental delay. For example, C-reactive protein, a potential marker of low-grade inflammation, was elevated only in children with PAE and a neurodevelopmental delay. Given that the results of this study are limited to a single time-point within the overall study, further validation in a longitudinal cohort across various populations would be beneficial.

The only recently published study to examine an immune outcome in adults with PAE found significantly higher rates of infections complicating pregnancy in women with FAS (37%) relative to a comparison group of pregnant women without FAS (7%).^48^ Overall, there is a notable gap in clinical literature examining immune function in individuals with PAE/FASD of any age. Existing clinical evidence, while variable, suggests that PAE may compromise immune function and contribute to susceptibility to infection and inflammatory conditions across the life span. Few studies have addressed autoimmune disease in this population, and the impact of this potential immune dysfunction on the daily lives of individuals with PAE remains largely unexplored. Additionally, it would be beneficial to further explore the impact of environmental factors (e.g., access to pediatric care) on the immune health of individuals with PAE.

### Reproductive Health

The FASD Changemakers Health Survey reported a number of reproductive health problems in adolescents and adults with FASD.^26^ Notably, 11% of females with FASD reported recurrent miscarriage, which is almost six times higher than rates seen within the general population. Adolescent and adult females with FASD also had high rates of premature menopause (7%), with menopause commencing as early as adolescence (under age 20) in some respondents. Among adolescent and adult males with FASD, 5% reported undescended testes. However, the rates of dysmenorrhea, ovarian cysts, infertility, and pregnancies resulting in preterm birth were similar in adolescents and adults with FASD as in the general population.^26^ A systematic review by Akison et al.^20^ (*n* = 5 clinical studies) revealed the scarcity of research examining reproductive outcomes in individuals with PAE/FASD. The available clinical evidence suggests that PAE may delay puberty onset in both male and female children/adolescents. In males, PAE may also alter testosterone levels, impacting testes development and sperm quality. There was greater preclinical evidence available (*n* = 18 studies), which demonstrated the effects of PAE on both male and female reproductive health.^20^

There were no recent clinical studies examining reproductive health in males with PAE. There were also no recent clinical studies exploring puberty, the menstrual cycle, fertility/ovarian parameters, miscarriage, menopause, or other gynecological health problems in females with PAE. The only recent clinical article on reproductive health assessed pregnancy complications and perinatal outcomes in women with and without FAS.^48^ Schellenberg et al.^48^ analyzed 7.3 million medical and birth records and found that 23% of women with FAS experienced pre-existing or gestational hypertension (compared to 9% of women without FAS). Infants born to women with FAS were also more likely to be born prematurely (17%), be small for gestational age (26%), and require admission to the NICU (29%). These outcomes occurred at a significantly higher rate in infants born to women with FAS than in infants born to women in the non-FAS group, where the incidence rate was 5% to 9%.

Findings from recent preclinical rodent models can supplement the limited clinical evidence available on reproductive health.^50^–^52^ These studies examined factors relating to female reproductive health in offspring exposed to alcohol in utero, including ovarian parameters, adult reproductive hormones, and adult breeding performance. While low-moderate PAE (12.5% ethanol v/v diet ad libitum and 1 g of ethanol/kg body weight) had minimal effects on reproductive outcomes,^50^,^51^ heavy PAE (4 g of ethanol/kg body weight) was associated with abnormal ovarian parameters suggestive of premature ovarian insufficiency susceptibility in adulthood.^52^ Overall, the available evidence indicates that individuals with PAE/FASD may be at risk of reproductive health issues across the life span. As with other health domains, variations in PAE amount and timing, and in the extent of FASD presentation, likely play a crucial role in influencing these outcomes. There is a significant gap in understanding reproductive health in individuals with PAE/FASD, and further research into treatment strategies would be valuable. Given the high rates of genetic anomalies among individuals with FASD, it would also be beneficial to further explore the potential contributions of genetics, and other factors such as endocrine disorders, abnormal gonadal development, and epigenetics, in relation to fertility issues in individuals with PAE.^79^

### Vision and Hearing Impairments

The FASD Changemakers Health Survey reported high rates of hearing loss, developed either in childhood (15%) or later in life (8%).^26^ A systematic review and meta-analysis of the literature (*N* = 25 studies) showed that studies examining hearing impairments in children and adolescents (under age 18) with PAE/FASD remain limited and their findings variable.^21^ There was some evidence to suggest that functional hearing impairments, such as auditory processing difficulties and hearing loss, may be more prevalent in children and adolescents with FASD than in the general population.^21^,^80^ Similarly, the limited available evidence suggests that structural ear abnormalities (e.g., microtia) may also be more common among children with PAE/FASD.^21^

Two recently published articles by Simões et al.^53^,^54^ examined auditory outcomes in adolescents (ages 13 to 14) with PAE but without the phenotypic facial features associated with FAS/partial FAS. Although the prevalence of hearing difficulties tended to be slightly higher in the exposed group relative to a TD comparison group, there was no significant difference in measures of auditory processing.^53^ Interestingly, electroencephalography found that the brain activity of this group of adolescents with PAE responded differently to rare or unexpected sounds when compared to TD controls. This suggests that even in the absence of overt differences in hearing and auditory processing, adolescents with PAE may still exhibit different cortical patterns related to detecting and responding to auditory stimuli.^54^

Similarly, the FASD Changemakers Health Survey documented high rates of eye abnormalities and vision problems in adolescents and adults with FASD.^26^ In this survey, 9% to 65% of respondents reported some type of eye-related issue, such as refractive errors, a need for glasses, and strabismus.^26^ A systematic review and meta-analysis (*N* = 35 studies) examined a variety of vision impairments in children and adolescents (under age 18) with PAE/FASD.^22^ The analyses reported that functional eye abnormalities, such as subnormal visual acuity and strabismus, may be more prevalent in children with FASD. Structural eye abnormalities, such as abnormal retinal vessel tortuosity and optic nerve hypoplasia, may also be more common in this population.^22^

Recent clinical studies on vision in individuals with PAE/FASD have examined a range of these previously reported eye abnormalities.^27^,^55^–^59^ A recent caregiver questionnaire emphasized the high prevalence of vision impairments in children and adolescents with FASD, with 45% clinically diagnosed with an eye condition.^27^ Although some studies examining visual acuity in children with FASD reported results within a normal range,^55^,^56^ comparisons to TD children revealed significantly “poorer” visual acuity in the FASD group.^56^ Varying degrees of eye misalignment, such as strabismus (or heterotropia), were commonly observed across studies,^27^,^56^–^58^ and measures of visual perception also indicated a high prevalence of impairment in this population.^56^,^57^ Recent studies examining eye function beyond standard vision assessment were less common, but Pueyo et al.^59^ reported evidence of poorer oculomotor control in children with FASD when compared to TD controls.

Gyllencreutz et al.^57^ conducted one of the few studies to investigate eye abnormalities in adults with FASD (ages 19 to 28). Notably, this study was a prospective follow-up with participants who had undergone an ophthalmological examination in childhood. The analyses found that rates of abnormal ophthalmic outcomes, such as refractive errors, heterotropia, subnormal stereoacuity, and structural eye abnormalities, remained stable or increased with age. This indicates that eye abnormalities commonly reported in children with FASD likely persist and may potentially worsen further into adulthood.

Not all recently published articles found notable differences in ophthalmological findings between children with PAE/FASD and children in the comparison group.^55^,^58^ While study design may play a role in some instances (e.g., reliance on medical records instead of purposeful recruitment), the variable findings also highlight the heterogeneity of eye abnormalities in individuals with FASD. There is insufficient understanding of how visual impairments may be influenced by ethnicity and environmental factors, which Tsang et al.^55^ postulate may explain their reported low rates of eye abnormalities in a remote Aboriginal Australian population with high rates of PAE. Such factors may also apply in the context of hearing outcomes, but literature remains sparse. Overall, the existing literature on hearing/vision problems in individuals with PAE suggests that ear and eye abnormalities may be common in this population. Despite limited studies in adulthood, hearing and vision problems are likely to persist across the life span. The impact of these impairments on the quality of life of children and adults with FASD, as well as the effectiveness of intervention strategies, remains unclear.

### Sleep Disturbances

The FASD Changemakers Health Survey reported high rates of sleep problems in adolescents and adults with FASD, with 40% to 70% of respondents describing some degree of sleep disturbances.^26^ The presence of persistent sleep problems in individuals with FASD is largely consistent across studies.^81^–^83^ A systematic review (*n* = 11 studies) reported that 55% to 85% of children with PAE/FASD had sleep disturbances relating to delayed sleep onset and frequent night wakings.^23^ Existing reviews also suggest that objective measures of sleep (e.g., actigraphy and polysomnography) tend to show similarly disturbed sleep patterns in individuals with PAE/FASD.^81^–^83^

Five recently published studies assessed sleep through caregiver reports.^60^–^64^ Chandler et al.^60^ reported frequent insomnia symptoms (40% to 45%) and nightmares (38% to 74%) in Australian children with PAE/FASD (ages 3 to 10). Similarly, other studies found a high prevalence of caregiver-reported delayed sleep onset and frequent night wakings in children and adolescents with PAE/FASD (ages 3 to 17).^61^–^63^ When compared to TD controls, these measures were significantly higher in children and adolescents with PAE/FASD.^61^,^62^ Adolescents with FASD (ages 11 to 17) also had high rates of insomnia symptoms (56%) and difficulty falling asleep (77%).^64^

Objective measures of sleep are less commonly used in studies due to challenges relating to access and cost of specialized equipment, as well as sensory and behavioral differences in individuals with PAE/FASD. Two recent studies used actigraphy to examine wake-sleep patterns in children and adolescents with FASD.^61^,^62^ Benson et al.^61^ found that children and adolescents with FASD (ages 5 to 16) had approximately 1 hour less sleep than their TD counterparts but found no differences in sleep onset or night wakings. Similarly, Inkelis et al.^62^ found no difference in mean values of sleep parameters between children and adolescents (ages 6 to 17) with and without heavy PAE (four or more drinks per occasion at least once per week, or 14 or more drinks per week); however, the study did report greater variability in sleep patterns on a night-to-night basis in the PAE group. In both of these studies, actigraphy results did not entirely reflect caregiver-reported sleep disturbances. This discrepancy may stem from subjective biases in caregiver reporting and the fact that actigraphy can only provide a snapshot of a child’s sleeping habits. The discrepancy likely is further compounded by actigraphy’s low specificity for detecting wakefulness and brief or subtle night wakings.^84^,^85^ Gold-standard polysomnography data is limited in this population, and research assessing sleep problems in adults is scarce.

Additional points of interest included evidence of sleep-disordered breathing (e.g., sleep apnea) in some individuals with PAE/FASD, potentially due to PAE-related neurological impairments and structural abnormalities.^61^–^64^ Daytime tiredness also has frequently been reported, with some research linking sleep disturbances to daytime impairments in areas such as executive function and working memory.^60^,^63^,^64^ There is also growing evidence to suggest that environmental factors and negative life experiences may exacerbate existing sleep problems in children with PAE/FASD.^65^,^66^ Overall, literature on sleep in individuals with PAE/FASD depicts a pattern of persistent sleep problems, which likely have a carryover effect on other aspects of daily life. Despite a lack of studies examining sleep in adults with FASD, the lived experiences reported in the FASD Changemakers Health Survey suggests that these sleep disturbances may persist across the life span.^26^

## Discussion and Conclusions

### Summary of Findings

**Figure 2:**
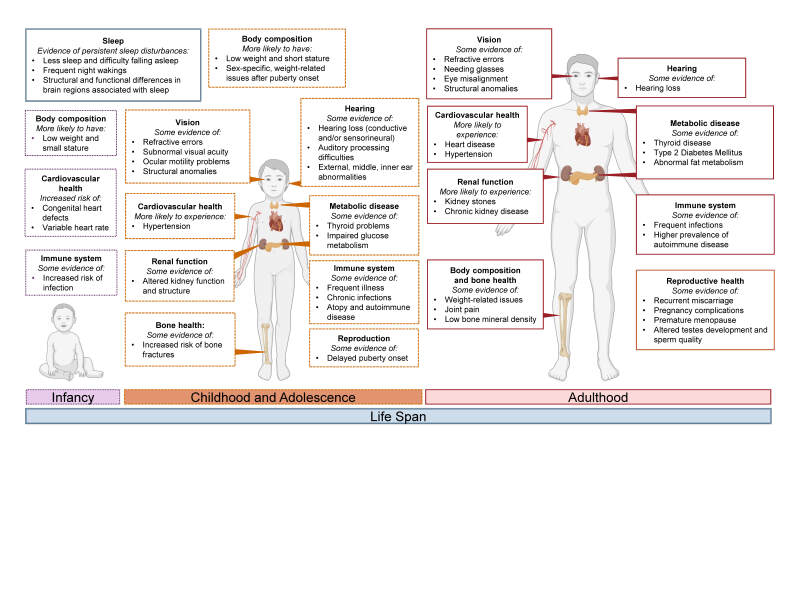
Summary of physical health challenges experienced by some individuals with PAE/FASD across the life span Evidence suggests that individuals with PAE/FASD may experience challenges related to sleep; vision; hearing; metabolism; body composition; and bone, cardiovascular, renal, immune, and/or reproductive health. Commonly reported challenges from different life stages are shown, including infancy (purple, dotted lines), childhood and adolescence (dark orange, dashed lines), and adulthood (red, solid lines). Dark blue double lines represent the life span. Image created using BioRender.com.

This narrative review aimed to examine the literature on the whole-body effects of PAE across the life span, with a focus on summarizing the latest scientific evidence underpinning the self-reported physical health problems of individuals with FASD documented through the FASD Changemakers Health Survey. A summary of the whole-body domains explored in this review and the health problems reported in the available literature are presented in Figure 2. Overall, existing evidence suggests that individuals with PAE/FASD may be more likely to have comorbidities associated with metabolic disease; body composition; cardio-renal, immune, and/or reproductive health; as well as sleep difficulties and vision/hearing impairments. The lived experiences of adolescents and adults with FASD, alongside the available preclinical and clinical evidence, highlight the importance of recognizing, monitoring, and supporting the health of those with FASD in its entirety, beyond standard clinical features relating to the brain, behavior, and mental health.^26^

### Limitations of the Review

Although the current review was able to integrate the findings of existing systematic reviews and explore the latest literature on these topics to offer a comprehensive picture of the whole-body effects of PAE across the life span, the human body is complex and its systems are numerous. Therefore, not all health difficulties reported by adults with FASD in the FASD Changemakers Health

Survey were explored.^26^ This included areas relating to oral health and sensory difficulties. The complexities of these health challenges have been recently summarized across a number of systematic reviews.^1^,^23^,^86^,^87^ Additionally, it was challenging to thoroughly discuss every aspect of each health domain and examine other areas of health reported in the literature but not explored in the FASD Changemakers Health Survey. For instance, the endocrine domain was limited to specific metabolic diseases and did not address evidence examining potential differences in hypothalamic-pituitary axis activity in this population.^88^–^91^ Finally, this narrative review largely focused on the clinical evidence, drawing on preclinical research only in particular areas where clinical research was significantly lacking. Expanding the scope to include more preclinical evidence and qualitative research would be beneficial in providing a deeper understanding of biological mechanisms and patient experiences.

### Gaps in the Literature and Future Directions

**Figure 3:**
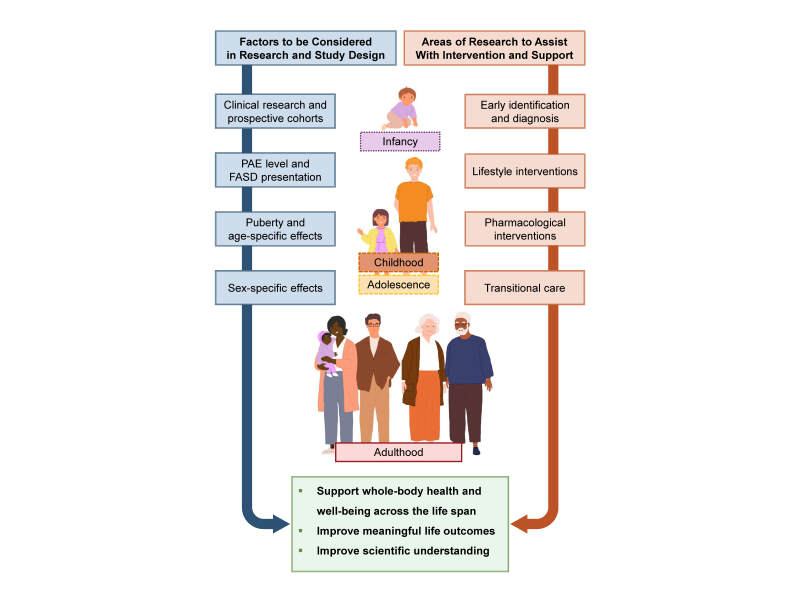
Potential future directions to improve scientific understanding and support the health and well-being of individuals with PAE/FASD across the life span Factors to be considered in research and study design are depicted on the left (dark blue, double-lined). More clinical research will be valuable, especially prospective pregnancy cohorts with long-term follow-ups. Disaggregation of data based on factors such as age, sex, and PAE/FASD level can further increase understanding of the impact of PAE on physical health outcomes. Areas of research relating to interventions and support are depicted on the right (dark orange, double-lined). Suggested areas include studies encouraging early identification of FASD and physical health challenges as well as assisting individuals with FASD as they transition from adolescence to adulthood. Studies that explore lifestyle and pharmacological interventions to ensure they are safe, applicable, and effective also would be valuable. Lifestyle interventions may offer a holistic approach to supporting the overall health and well-being of individuals with PAE/FASD. Illustrations adapted from Canva.

The existing evidence raises the question of what is needed to improve scientific understanding in this area and to support the health and well-being of individuals with PAE/FASD across the life span. Several key factors relating to future research and study design, as well as areas to assist in intervention and support, are summarized in Figure 3.

Many health domains were lacking in clinical evidence, limiting current understanding of how PAE may contribute to poor health outcomes and chronic disease in humans. This was particularly apparent in areas such as reproductive health. Additionally, how these health challenges may evolve across the life span and present in adults with FASD remains underexplored, underscoring the importance of potential further research using prospective pregnancy cohorts with long-term follow-ups. Notably, while this article was under review, Coles et al.^92^ published a study exploring self-reported adult physical health in individuals from a longitudinal PAE cohort. The study’s findings provide further support for the long-term physical health challenges that some individuals with PAE/FASD can experience. Additionally, there also tended to be a lack of consideration of participant characteristics such as age and biological sex, particularly regarding their effects after the onset of puberty. These characteristics may have an impact on health outcomes and should therefore be considered in study design, and data should be analyzed accordingly where possible.^30^,^41^,^43^,^74^ It would also be beneficial to ensure appropriate comparison groups are used and matched to minimize confounding variables (e.g., socioeconomic factors) and provide insight into health outcomes associated with PAE.^30^,^41^,^43^,^74^

Current literature also is frequently restricted to a single cohort of individuals described as having “PAE” or “FASD” and often lacks detailed information about PAE levels in both study and comparison groups. The level and timing of PAE can play a major role in the extent of physical, cognitive, and behavioral differences observed, with recent clinical articles reporting dose-dependent effects on some physical outcomes (e.g., birth weight and renal function).^1^,^43^,^44^ Accurate PAE levels are needed to examine the possibility of dose-response relationships across a wider range of physical health outcomes; alternatively, if accurate PAE data is lacking, future research could consider the degree of FASD presentation.

Additionally, few studies have examined PAE alongside other prenatal and postnatal risk factors. This is a critical area for future research, because these secondary factors may play a significant role in the development or exacerbation of adverse physical health outcomes in this population. However, research typically adopts a “single exposure” approach that does not consider the complex external (e.g., social, cultural, environmental) and internal (e.g., metabolism, inflammation) factors surrounding PAE. The concept of the “exposome,” which aims to be a cumulative measure of these environmental exposures from conception onward, could advance our understanding by providing a holistic view of all exposures, including prenatal and postnatal adversity across the life span.^93^,^94^ Better understanding the consequences of PAE through an exposome lens may provide novel insights into risk and resilience, informing personalized interventions to optimize health outcomes. Overall, future research could disaggregate data based on PAE/FASD level and consider a wider range of associated prenatal and postnatal factors where possible.

This narrative review highlights the importance of future research relating to intervention and support services. As seen in Figure 3, this could include research promoting early identification of FASD and related physical health challenges, as well as studies aimed at assisting individuals with FASD as they transition from adolescence to adulthood.^15^ Additionally, further studies examining pharmacological interventions and nutritional supplements could help determine the effectiveness of these approaches in addressing potential physical health problems.^95^ For instance, therapeutic agents that target oxidative stress and inflammation may support whole-body health.^96^–^98^ It is equally important to approach the use of psychotropic medications with care, because these agents can have significant side effects on various aspects of physical health, especially in children and adolescents.^95^,^99^

Research exploring lifestyle interventions could be valuable. Targeting diet, physical activity, and/or sleep may offer a significant opportunity to advance the overall health and well-being of individuals with PAE/FASD. To our knowledge, only one research group has examined physical activity-based interventions in children with FASD, and studies investigating sleep and diet-focused interventions have only assessed the effects of nutritional supplementation.^15^,^100^–^103^ There is a reasonable body of research from other neurodevelopmental conditions that could inform further research on intervention strategies for individuals with FASD.^15^,^104^–^107^ Lifestyle interventions targeting these areas have the potential to benefit both cognitive and physical health. These interventions could be particularly meaningful for individuals with PAE/FASD and offer a holistic approach to health and well-being. It would be beneficial for these types of interventions to consider social determinants of health, because these factors impact access to nutritious foods and environments that support quality sleep and meaningful physical activity.

Interest in physical health outcomes in individuals with PAE/FASD has been growing. However, there continues to be a lack of clinical research addressing the whole-body health of this population. Future research that embraces a holistic approach to understanding the complex interactions between prenatal and postnatal factors influencing health outcomes could provide critical opportunities to implement interventions to improve the health and well-being of individuals with PAE/FASD across the life span.

**KEY TAKEAWAYS**
- Recent clinical evidence integrated with the lived experiences of adolescents and adults with fetal alcohol spectrum disorder (FASD) highlights the range of physical health challenges people can experience.
- Recent research suggests that comorbid physical health conditions may be associated with PAE across the life span for individuals with FASD. These conditions include metabolic disorders; body composition; cardiac, renal, reproductive, and immune health; as well as hearing/vision and sleep.
- Recent research highlights the importance of recognizing, monitoring, and supporting the health of individuals with FASD, beyond the standard clinical features relating to the brain and behavior.
- The review identifies emerging areas of concern relating to the physical health of individuals with FASD that require further research and highlights key considerations for future study design.

## Figures and Tables

**Table 1:** Literature Search Details

Health Domain	Search Terms	Phase One Most Recent Relevant Review
PAE and FASD	prenatal alcohol OR prenatal ethanol OR fetal alcohol OR fetal alcohol OR fetal alcohol spectrum disorder OR fetal alcohol spectrum disorder OR fetal alcohol syndrome OR fetal alcohol syndrome OR partial fetal alcohol syndrome OR partial fetal alcohol syndrome OR alcohol-related neurodevelopmental disorder OR alcohol related neurodevelopmental disorder OR alcohol-related birth defects OR alcohol related birth defects OR static encephalopathy OR neurobehavioral disorder alcohol exposed OR neurobehavioral disorder associated with prenatal alcohol exposure	-
Metabolism	metabol* OR endocrine OR diabetes OR thyroid	Akison et al.^17^
Body Composition	body composition OR obesity OR weight OR bone OR skeletal OR joint	Akison et al.^17^
Cardio-Renal	cardi* OR heart OR renal OR kidney	Reid et al.^18^
Immune	immun* OR autoimmun* OR infection OR allergy	Reid et al.^19^
Reproductive	reproduct* OR fertility	Akison et al.^20^
Hearing	face OR ear OR hearing OR auditory	Cheung et al.^21^
Vision	eye OR sight OR vision OR opthalm*	Tsang et al.^22^
Sleep	sleep OR circadian rhythm	Reid et al.^23^

**Table 2:** Number of Studies Included and Excluded for Each Health Domain in Phase Two of the Literature Search

Health Domain	Initial Search	Duplicates	Title-Abstract Review	Exclusion				Full-Text Review	Exclusion			Other Additions*	Final Inclusion
				Wrong Art icle Type	Not PAE/FASD	Irrelevant Topic	Duplicates		Wrong Article Type	Not PAE/FASD	Irrelevant Topic		
Metabolism	260	108	152	3	19	104	-	26	5	-	15	-	6
Body Composition	278	103	175	2	5	120	1	47	1	-	40	1	7
Cardio-Renal Function	156	67	89	-	6	45	-	38	11	-	19	2	10
Immune System	207	85		1	14	90	-	17	2	-	11	2	6
Reproduction	52	22	30	-	5	19	-	6	1	-	1	-	4
Hearing	50	18	32	2	-	22	-	8	1	1	4	-	2
Vision	68	24	44	1	-	27	1	15	2	1	6	-	6
Sleep	31	10	21	-	-	8	1	12	3	-	2	-	7
Total	1,102	437	665	9	49	435	3	169	26	2	98	5	48
												Duplicate	7
												Total Articles	41

* Two studies were identified from the reference lists of other literature and three were previously excluded from other health domain screenings but were relevant to other areas explored.

*Note*: Individual searches and screenings were conducted for each health domain.

## References

[1] Akison LK, Hayes N, Vanderpeet C (2024). Prenatal alcohol exposure and associations with physical size, dysmorphology and neurodevelopment: A systematic review and meta-analysis. BMC Med.

[2] American Psychiatric Association (2022). Diagnostic and Statistical Manual of Mental Disorders.

[3] Banerji A, Shah C (2017). Ten-year experience of fetal alcohol spectrum disorder; diagnostic and resource challenges in Indigenous children. Paediatr Child Health.

[4] Kambeitz C, Klug MG, Greenmyer J, Popova S, Burd L (2019). Association of adverse childhood experiences and neurodevelopmental disorders in people with fetal alcohol spectrum disorders (FASD) and non-FASD controls. BMC Pediatr.

[5] Sessa F, Salerno M, Esposito M (2022). Understanding the relationship between fetal alcohol spectrum disorder (FASD) and criminal justice: A systematic review. Healthcare.

[6] Astley SJ (2013). Validation of the fetal alcohol spectrum disorder (FASD) 4-Digit Diagnostic Code. J Popul Ther Clin Pharmacol.

[7] Hoyme HE, Kalberg WO, Elliott AJ (2016). Updated clinical guidelines for diagnosing fetal alcohol spectrum disorders. Pediatrics.

[8] Kable JA, Mukherjee RAS (2017). Neurodevelopmental disorder associated with prenatal exposure to alcohol (ND-PAE): A proposed diagnostic method of capturing the neurocognitive phenotype of FASD. Eur J Med Genet.

[9] Lange S, Probst C, Gmel G, Rehm J, Burd L, Popova S (2017). Global prevalence of fetal alcohol spectrum disorder among children and youth: A systematic review and meta-analysis. JAMA Pediatr.

[10] Popova S, Lange S, Shield K, Burd L, Rehm J (2019). Prevalence of fetal alcohol spectrum disorder among special subpopulations: A systematic review and meta-analysis. Addiction.

[11] Chung DD, Pinson MR, Bhenderu LS, Lai MS, Patel RA, Miranda RC (2021). Toxic and teratogenic effects of prenatal alcohol exposure on fetal development, adolescence, and adulthood. Int J Mol Sci.

[12] Coles CD, Gailey AR, Mulle JG, Kable JA, Lynch ME, Jones KL (2016). A comparison among 5 methods for the clinical diagnosis of fetal alcohol spectrum disorders. Alcohol Clin Exp Res.

[13] Popova S, Lange S, Shield K (2016). Comorbidity of fetal alcohol spectrum disorder: A systematic review and meta-analysis. Lancet.

[14] Chu JTW, McCormack J, Marsh S, Wells A, Wilson H, Bullen C (2022). Impact of prenatal alcohol exposure on neurodevelopmental outcomes: A systematic review. Health Psychol Behav Med.

[15] Abdul-Rahman OA, Petrenko CLM (2023). Fetal Alcohol Spectrum Disorders: A Multidisciplinary Approach.

[16] Greenmyer JR, Klug MG, Kambeitz C, Popova S, Burd L (2018). A multicountry updated assessment of the economic impact of fetal alcohol spectrum disorder: Costs for children and adults. J Addict Med.

[17] Akison LK, Reid N, Wyllie M, Moritz KM (2019). Adverse health outcomes in offspring associated with fetal alcohol exposure: A systematic review of clinical and preclinical studies with a focus on metabolic and body composition outcomes. Alcohol Clin Exp Res.

[18] Reid N, Akison LK, Hoy W, Moritz KM (2019). Adverse health outcomes associated with fetal alcohol exposure: A systematic review focused on cardio-renal outcomes. J Stud Alcohol Drugs.

[19] Reid N, Moritz KM, Akison LK (2019). Adverse health outcomes associated with fetal alcohol exposure: A systematic review focused on immune-related outcomes. Pediatr Allergy Immunol.

[20] Akison LK, Moritz KM, Reid N (2019). Adverse reproductive outcomes associated with fetal alcohol exposure: A systematic review. Reproduction.

[21] Cheung MMY, Tsang TW, Watkins R, Birman C, Popova S, Elliott EJ (2022). Ear abnormalities among children with fetal alcohol spectrum disorder: A systematic review and meta-analysis. J Pediatr.

[22] Tsang TW, Finlay-Jones A, Perry K (2023). Eye abnormalities in children with fetal alcohol spectrum disorders: A systematic review. Ophthal Epidemiol.

[23] Reid N, Kent N, Hewlett N (2023). Factors to be considered as part of a holistic assessment for fetal alcohol spectrum disorder: A scoping review. Alcohol Clin Exp Res.

[24] Caputo C, Wood E, Jabbour L (2016). Impact of fetal alcohol exposure on body systems: A systematic review. Birth Defects Res C Embryo Today.

[25] Barker DJP (2007). The origins of the developmental origins theory. J Intern Med.

[26] Himmelreich M, Lutke CJ, Hargrove ET, Begun AL, Murray MM (2020). The lay of the land: Fetal alcohol spectrum disorder (FASD) as a whole-body diagnosis. The Routledge Handbook of Social Work and Addictive Behaviors.

[27] Reid N, Hayes N, Young SB, Akison LK, Moritz KM (2021). Caregiver-reported physical health status of children and young people with fetal alcohol spectrum disorder. J Dev Orig Health Dis.

[28] Kable JA, Mehta PK, Coles CD (2021). Alterations in insulin levels in adults with prenatal alcohol exposure. Alcohol Clin Exp Res.

[29] Weeks O, Bossé GD, Oderberg IM (2020). Fetal alcohol spectrum disorder predisposes to metabolic abnormalities in adulthood. J Clin Invest.

[30] Flannigan K, Poole N, Cook J, Unsworth K (2023). Sexrelated differences among individuals assessed for fetal alcohol spectrum disorder in Canada. Alcohol Clin Exp Res.

[31] Bake S, Pinson MR, Pandey S (2021). Prenatal alcohol-induced sex differences in immune, metabolic and neurobehavioral outcomes in adult rats. Brain Behav Immun.

[32] Nguyen TMT, Steane SE, Moritz KM, Akison LK (2019). Prenatal alcohol exposure programmes offspring disease: Insulin resistance in adult males in a rat model of acute exposure. J Physiol.

[33] Barry CV, Chrysanthopoulou SA, Tallo V (2024). The impact of prenatal alcohol exposure on longitudinal growth, nutritional status, and insulin-like growth factor 1 in early childhood in Leyte, the Philippines. J Pediatr.

[34] Hayes N, Reid N, Akison LK, Moritz KM (2021). The effect of heavy prenatal alcohol exposure on adolescent body mass index and waist-to-height ratio at 12–13 years. Int J Obes (Lond).

[35] Beaulieu D, Treit S, Pagano JJ, Beaulieu C, Thompson R (2023). Cardiac magnetic resonance imaging in individuals with prenatal alcohol exposure. CJC Pediatr Congenit Heart Dis.

[36] Young SL, Gallo LA, Brookes DSK (2022). Altered bone and body composition in children and adolescents with confirmed prenatal alcohol exposure. Bone.

[37] Parviainen R, Auvinen J, Serlo W, Järvelin MR, Sinikumpu JJ (2020). Maternal alcohol consumption during pregnancy associates with bone fractures in early childhood. A birth-cohort study of 6718 participants. Bone.

[38] Hansen M, Greenop K, Yim D, Ramsay J, Thomas Y, Baynam GS (2021). Birth prevalence of congenital heart defects in Western Australia, 1990–2016. J Paediatr Child Health.

[39] Harvey DC, Baer RJ, Bandoli G, Chambers CD, Jelliffe-Pawlowski LL, Kumar SR (2022). Association of alcohol use diagnostic codes in pregnancy and offspring conotruncal and endocardial cushion heart defects. J Am Heart Assoc.

[40] Jolma LM, Koivu-Jolma M, Sarajuuri A, Torkki P, Autti-Rämö I, Sätilä H (2023). Children with FASD—Evolving patterns of developmental problems and intervention costs in ages 0 through 16 in Finland. Children.

[41] Cook JC, Lynch ME, Coles CD (2019). Association analysis: Fetal alcohol spectrum disorder and hypertension status in children and adolescents. Alcohol Clin Exp Res.

[42] Dyląg KA, Dumnicka P, Kowalska K, Migas-Majoch A, Przybyszewska K, Drożdż D (2024). Increased incidence of renal and urinary tract anomalies among individuals with fetal alcohol spectrum disorders (FASD). Birth Defects Res.

[43] Correia-Costa L, Schaefer F, Afonso AC (2020). Prenatal alcohol exposure affects renal function in overweight schoolchildren: Birth cohort analysis. Pediatr Nephrol.

[44] Das SK, McIntyre HD, Alati R, Al Mamun A (2019). Maternal alcohol consumption during pregnancy and its association with offspring renal function at 30 years: Observation from a birth cohort study. Nephrology (Carlton).

[45] Maxwell JR, DiDomenico J, Roberts MH (2024). Impact of low-level prenatal alcohol exposure and maternal stress on autonomic regulation. Pediatr Res.

[46] Vilcins D, Blake TL, Sly PD (2023). Effects of prenatal alcohol exposure on infant lung function, wheeze, and respiratory infections in Australian children. Alcohol Clin Exp Res.

[47] Bodnar TS, Raineki C, Wertelecki W (2020). Immune network dysregulation associated with child neurodevelopmental delay: Modulatory role of prenatal alcohol exposure. J Neuroinflammation.

[48] Schellenberg H, Baer RJ, Bandoli G (2024). Pregnancy characteristics and outcomes among birthing individuals with a diagnosis of fetal alcohol syndrome. Alcohol Clin Exp Res.

[49] Bodnar TS, Mak DY, Hill LA, Ellis L, Yu W, Weinberg J (2022). Modulatory role of prenatal alcohol exposure and adolescent stress on the response to arthritis challenge in adult female rats. EBiomedicine.

[50] McReight EK, Liew SH, Steane SE, Hutt KJ, Moritz KM, Akison LK (2020). Moderate episodic prenatal alcohol does not impact female offspring fertility in rats. Reproduction.

[51] Steane SE, Burgess DJ, Moritz KM, Akison LK (2024). The impacts of periconceptional alcohol on neonatal ovaries and subsequent adult fertility in the rat. Int J Mol Sci.

[52] Ni Y, Xu D, Lv F (2019). Prenatal ethanol exposure induces susceptibility to premature ovarian insufficiency. J Endocrinol.

[53] Simões HO, Massuda ET, Furtado EF, Zanchetta S (2023). Auditory outcomes in adolescents with prenatal alcohol exposure. Dev Neurosci.

[54] de Oliveira Simões H, Zanchetta S, Felipe Furtado E (2021). Differential cortical pattern in auditory task oddball paradigm in children exposed to alcohol during pregnancy. Neuroscience.

[55] Tsang TW, Allen T, Turner A (2024). Ophthalmic findings in aboriginal children with high rates of prenatal alcohol exposure and fetal alcohol spectrum disorder: The Lililwan project. Ophthalmic Epidemiol.

[56] Ayoub L, Aring E, Gyllencreutz E (2023). Visual and ocular findings in children with fetal alcohol spectrum disorders (FASD): Validating the FASD Eye Code in a clinical setting. BMJ Open Ophthalmol.

[57] Gyllencreutz E, Aring E, Landgren V, Svensson L, Landgren M, Grönlund MA (2020). Ophthalmologic findings in fetal alcohol spectrum disorders—A cohort study from childhood to adulthood. Am J Ophthalmol.

[58] Lyubasyuk V, Jones KL, Caesar MA, Chambers C (2023). Vision outcomes in children with fetal alcohol spectrum disorders. Birth Defects Res.

[59] Pueyo V, Castillo O, Gonzalez I (2020). Oculomotor deficits in children adopted from Eastern Europe. Acta Paediatr.

[60] Chandler-Mather N, Crichton A, Shelton D, Harris K, Donovan C, Dawe S (2025). Carer-reported sleep disturbance and carer- and teacher-rated executive functioning in children with prenatal alcohol exposure and fetal alcohol spectrum disorder. Child Neuropsychol.

[61] Benson AA, Mughal R, Dimitriou D, Halstead EJ (2023). Towards a distinct sleep and behavioural profile of fetal alcohol spectrum disorder (FASD): A comparison between FASD, autism and typically developing children. J Integr Neurosci.

[62] Inkelis SM, Soja J, Mattson SN, Chambers CD, Bhattacharjee R, Thomas JD (2024). Characteristics of sleep in children with heavy prenatal alcohol exposure. Alcohol Clin Exp Res.

[63] Gerstner T, Saevareid HI, Johnsen ÅR, Løhaugen G, Skranes J (2023). Sleep disturbances in Norwegian children with fetal alcohol spectrum disorders (FASD) with and without a diagnosis of attentiondeficit hyperactivity disorder or epilepsy. Alcohol Clin Exp Res.

[64] Hammond L, Joly V, Kapasi A (2022). Adaptive behavior, sleep, and physical activity in adolescents with fetal alcohol spectrum disorder. Res Dev Disabil.

[65] Chandler-Mather N, Betts J, Donovan C, Shelton D, Dawe S (2023). Understanding the impacts of childhood adversity on sleep problems in children with fetal alcohol spectrum disorder: A comparison of cumulative and dimensional approaches. Alcohol Clin Exp Res.

[66] Lund IO, Ystrom E (2022). Prenatal alcohol exposure and child sleep problems: A family-based quasi-experimental study. JCPP Adv.

[67] Chaker L, Razvi S, Bensenor IM, Azizi F, Pearce EN, Peeters RP (2022). Hypothyroidism. Nat Rev Dis Primers.

[68] Myrie SB, Pinder MA (2018). Skeletal muscle and fetal alcohol spectrum disorder. Biochem Cell Biol.

[69] Lunde ER, Washburn SE, Golding MC, Bake S, Miranda RC, Ramadoss J (2016). Alcohol-induced developmental origins of adult-onset diseases. Alcohol Clin Exp Res.

[70] Chen ZY, Li S, Guo LH, Peng X, Liu Y (2021). Prenatal alcohol exposure induced congenital heart diseases: From bench to bedside. Birth Defects Res.

[71] Dyląg KA, Anunziata F, Bandoli G, Chambers C (2023). Birth defects associated with prenatal alcohol exposure—A review. Children.

[72] Jurczyk M, Dyląg KA, Skowron K, Gil K (2019). Prenatal alcohol exposure and autonomic nervous system dysfunction: A review article. Folia Med Cracov.

[73] McDonnell P, Fornell P, Ponce S, Dyer L (2023). Baseline heart rate in infants with prenatal alcohol exposure: A systematic review and independent analysis. Birth Defects Res.

[74] Bake S, Rouzer SK, Mavuri S, Miranda RC, Mahnke AH (2023). The interaction of genetic sex and prenatal alcohol exposure on health across the lifespan. Front Neuroendocrinol.

[75] Oulerich Z, Sferruzzi-Perri AN (2024). Early-life exposures and long-term health: Adverse gestational environments and the programming of offspring renal and vascular disease. Am J Physiol Renal Physiol.

[76] Harambat J, Van Stralen KJ, Kim JJ, Tizard EJ (2012). Epidemiology of chronic kidney disease in children. Pediatr Nephrol.

[77] Gauthier TW (2015). Prenatal alcohol exposure and the developing immune system. Alcohol Res.

[78] Gauthier TW, Brown LAS (2017). In utero alcohol effects on foetal, neonatal and childhood lung disease. Paediatr Respir Rev.

[79] Douzgou S, Breen C, Crow YJ (2012). Diagnosing fetal alcohol syndrome: New insights from newer genetic technologies. Arch Dis Child.

[80] Simões HO, Zanchetta S, Furtado EF (2016). O que sabemos das alterações auditivas centrais em crianças expostas ao álcool na gestação? Revisão sistemática. CoDAS.

[81] Hanlon-Dearman A, Chen ML, Olson HC (2018). Understanding and managing sleep disruption in children with fetal alcohol spectrum disorder. Biochem Cell Biol.

[82] Inkelis SM, Thomas JD (2018). Sleep in infants and children with prenatal alcohol exposure. Alcohol Clin Exp Res.

[83] Wilson DA, Sullivan RM, Smiley JF, Saito M, Raineki C (2024). Developmental alcohol exposure is exhausting: Sleep and the enduring consequences of alcohol exposure during development. Neurosci Biobehav Rev.

[84] Van De Water ATM, Holmes A, Hurley DA (2011). Objective measurements of sleep for non-laboratory settings as alternatives to polysomnography—A systematic review. J Sleep Res.

[85] Martin JL, Hakim AD (2011). Wrist actigraphy. Chest.

[86] Bell JC, Raynes-Greenow C, Turner RM, Bower C, Nassar N, O’Leary CM (2014). Maternal alcohol consumption during pregnancy and the risk of orofacial clefts in infants: A systematic review and meta-analysis. Paediatr Perinat Epidemiol.

[87] Blanck-Lubarsch M, Dirksen D, Feldmann R, Hohoff A (2023). A systematic review: Facial, dental and orthodontic findings and orofacial diagnostics in patients with FASD. Front Pediatr.

[88] Das U, Gangisetty O, Chaudhary S (2023). Epigenetic insight into effects of prenatal alcohol exposure on stress axis development: Systematic review with meta-analytic approaches. Alcohol Clin Exp Res.

[89] Grimm J, Stemmler M, Golub Y (2021). The association between prenatal alcohol consumption and preschool child stress system disturbance. Dev Psychobiol.

[90] Podgórski R, Galiniak S, Mazur A, Domin A (2023). The association of the hypothalamic-pituitary-adrenal axis with appetite regulation in children with fetal alcohol spectrum disorders (FASDs). Nutrients.

[91] Bakhireva LN, Solomon E, Roberts MH (2024). Independent and combined effects of prenatal alcohol exposure and prenatal stress on fetal HPA axis development. Int J Mol Sci.

[92] Coles CD, Shapiro ZR, Kable JA, Stoner SA, Ritfeld GJ, Grant TM (2024). Prenatal alcohol exposure and health at midlife: Self-reported health outcomes in two cohorts. Alcohol Clin Exp Res.

[93] Wild CP (2012). The exposome: From concept to utility. Int J Epidemiol.

[94] Persson Waye K, Löve J, Lercher P (2023). Adopting a child perspective for exposome research on mental health and cognitive development—Conceptualisation and opportunities. Environ Res.

[95] Ritfeld GJ, Kable JA, Holton JE, Coles CD (2022). Psychopharmacological treatments in children with fetal alcohol spectrum disorders: A review. Child Psychiatry Hum Dev.

[96] Zhang Y, Wang H, Li Y, Peng Y (2018). A review of interventions against fetal alcohol spectrum disorder targeting oxidative stress. Int J Dev Neurosci.

[97] Forman HJ, Zhang H (2021). Targeting oxidative stress in disease: Promise and limitations of antioxidant therapy. Nat Rev Drug Discov.

[98] Furman D, Campisi J, Verdin E (2019). Chronic inflammation in the etiology of disease across the life span. Nat Med.

[99] Abosi O, Lopes S, Schmitz S, Fiedorowicz JG (2018). Cardiometabolic effects of psychotropic medications. Horm Mol Biol Clin Investig.

[100] Keiver K, Bertram CP, Orr AP, Clarren S (2015). Salivary cortisol levels are elevated in the afternoon and at bedtime in children with prenatal alcohol exposure. Alcohol.

[101] Pritchard Orr AB, Keiver K, Bertram CP, Clarren S (2018). FAST Club: The impact of a physical activity intervention on executive function in children with fetal alcohol spectrum disorder. Adapt Phys Activ Q.

[102] Akison LK, Kuo J, Reid N, Boyd RN, Moritz KM (2018). Effect of choline supplementation on neurological, cognitive, and behavioral outcomes in offspring arising from alcohol exposure during development: A quantitative systematic review of clinical and preclinical studies. Alcohol Clin Exp Res.

[103] Andreu-Fernández V, La Maida N, Marquina M (2024). Novel interventions on comorbidities in patients with fetal alcohol spectrum disorder (FASD): An integrative review. Biomedicines.

[104] Li Y, Feng Y, Zhong J (2023). The effects of physical activity interventions in children with autism spectrum disorder: A systematic review and network meta-analysis. Rev J Autism Dev Disord.

[105] Welsch L, Alliott O, Kelly P, Fawkner S, Booth J, Niven A (2021). The effect of physical activity interventions on executive functions in children with ADHD: A systematic review and meta-analysis. Ment Health Phys Act.

[106] Svensson L, Stylianou M, Hill J, Trost SG, Cairney J (2024). An acceptability and feasibility investigation of a community-based motor program for autistic children with moderate and high support needs. Res Autism Spec Disord.

[107] Rigney G, Ali NS, Corkum PV (2018). A systematic review to explore the feasibility of a behavioural sleep intervention for insomnia in children with neurodevelopmental disorders: A transdiagnostic approach. Sleep Med Rev.

